# A Histopathological Scheme for the Quantitative Scoring of Intervertebral Disc Degeneration and the Therapeutic Utility of Adult Mesenchymal Stem Cells for Intervertebral Disc Regeneration

**DOI:** 10.3390/ijms18051049

**Published:** 2017-05-12

**Authors:** Cindy C. Shu, Margaret M. Smith, Susan M. Smith, Andrew J. Dart, Christopher B. Little, James Melrose

**Affiliations:** 1 Raymond Purves Bone and Joint Research Laboratory, Kolling Institute, Northern Sydney Local Health District, St. Leonards, NSW 2065, Australia; Cindy.shu@sydney.edu.au (C.C.S.); mobsmith@sydney.edu.au (M.M.S.); Susan.smith@sydney.edu.au (S.M.S.); christopher.little@sydney.edu.au (C.B.L.); 2 Faculty of Veterinary Science, University Veterinary Teaching Hospital, University of Sydney, Camden, NSW 2050, Australia; andrew.dart@sydney.edu.au; 3 Sydney Medical School, Northern, The University of Sydney, Royal North Shore Hospital, St. Leonards, NSW 2065, Australia; 4 Graduate School of Biomedical Engineering, University of New South Wales, Kensington, NSW 2052, Australia

**Keywords:** histopathology scoring, AF, disc degeneration, IVD, quantitative histology

## Abstract

The purpose of this study was to develop a quantitative histopathological scoring scheme to evaluate disc degeneration and regeneration using an ovine annular lesion model of experimental disc degeneration. Toluidine blue and Haematoxylin and Eosin (H&E) staining were used to evaluate cellular morphology: (i) disc structure/lesion morphology; (ii) proteoglycan depletion; (iii) cellular morphology; (iv) blood vessel in-growth; (v) cell influx into lesion; and (vi) cystic degeneration/chondroid metaplasia. Three study groups were examined: 5 × 5 mm lesion; 6 × 20 mm lesion; and 6 × 20 mm lesion plus mesenchymal stem cell (MSC) treatment. Lumbar intervertebral discs (IVDs) were scored under categories (i–vi) to provide a cumulative score, which underwent statistical analysis using STATA software. Focal proteoglycan depletion was associated with 5 × 5 mm annular rim lesions, bifurcations, annular delamellation, concentric and radial annular tears and an early influx of blood vessels and cells around remodeling lesions but the inner lesion did not heal. Similar features in 6 × 20 mm lesions occurred over a 3–6-month post operative period. MSCs induced a strong recovery in discal pathology with a reduction in cumulative histopathology degeneracy score from 15.2 to 2.7 (*p* = 0.001) over a three-month recovery period but no recovery in carrier injected discs.

## 1. Introduction

The intervertebral disc (IVD) is a tough but intricately organized connective tissue which resists tension and weight bearing during axial and torsional spinal loading but also provides spinal stability and flexibility [1]. The IVD achieves these remarkable properties through the interplay of a number of connective tissues of disparate structure and function that collectively endow the composite IVD with its unique mechanical properties [2,3]. The outer regions of the IVD, the annulus fibrosus (AF), is composed of collagen rich annular lamellae, which are arranged around a central proteoglycan rich region called the nucleus pulposus (NP). Superior and inferior endplates of hyaline cartilage, the cartilaginous endplates (CEPs), enclose the NP where it borders the spinal vertebrae. Collectively, this arrangement of connective tissues equips the IVD with unique weight-bearing capability [4]. The hydration provided by the hydrophilic proteoglycans of the NP afford viscoelastic and hydrodynamic properties which equip the IVD with its ability to act as a weight bearing cushion [5]. The major proteoglycan of the NP is a large chondroitin sulphate (CS)and keratan sulphate (KS)rich proteoglycan of the Hyalectan family called aggrecan [5,6,7,8]. Hyaluronan forms massive link-protein stabilized aggregates with aggrecan which have impressive water regain properties; the high concentration of aggrecan in the NP generate an internal hydrostatic pressure in the IVD which counters axial spinal loading [9]. Furthermore, bulging of the NP upon axial spinal loading results in load transfer to the annular lamellae which bulge and generate radial hoop stresses which dissipate shear stresses generated during axial loading of the spine. Type I collagen is the major fibrillar collagen of the AF with its concentration maximal in the outer annular lamellae. Type II collagen is also present in the AF but to a far lesser extent, the NP however is rich in type II collagen where type II fibrillar networks entrap the hyaluronan (HA)-aggrecan-link ternary complexes which are so important for discal biomechanical function [8,9,10,11]. Other, more minor, proteoglycans with specific roles to play in the composite IVD include members of the small leucine repeat proteoglycan (SLRP) family, decorin, biglycan, fibromodulin, lumican, and keratocan [12,13], the CS-hyalectan member, versican, the lubricative proteoglycan, lubricin, and the large modular HS/CS proteoglycan perlecan [14] (reviewed in [15]). With ageing and degeneration, proteoglycans are lost from the NP through proteolytic cleavage [16,17,18,19] and the IVD becomes progressively more dehydrated and less biomechanically competent [3]. The matrix metalloproteinases (MMPs) produced by disc cells are regulated by the mechanical microenvironment of the cells [17,18,20] and can predispose the IVD to further mechanical damage and the generation of a number of characteristic lesions primarily in the AF [21]. The loss of aggrecan from the IVD lessens its ability to swell and act as a weight-bearing cushion and traumatic mechanical damage to the NP may be irreversible, distinguishing degenerative disc disease (DDD) from normal aging of the IVD [22]. Loss of discal aggrecan also promotes angiogenesis, and may lead to the ingrowth of blood vessels and nociceptive nerves and perception of discogenic low back pain (LBP) [23,24].

Degenerative disc disease (DDD) is now recognized as a major global disorder which along with associated low back pain (LBP) will be of sufficient severity to warrant consultation with a physician and will affect 80% of the general population some time in their lifetime [25]. Unfortunately, there are no effective treatments available to treat this condition other than surgical intervention. DDD results in the generation of LBP in the biomechanically incompetent IVD and has major socioeconomic consequences (reviewed in [26]). In the last two decades, a number of biological agents have been evaluated as prospective therapeutic interventions for DDD and this has led to the identification of several promising therapeutic targets [27,28,29]. These compounds have the ability to induce cellular proliferation, matrix deposition, regulate MMP production and activation, regulate inflammation and modify vascular in-growth and cell viability [30]. HA oligosaccharides of 10–12 saccharide units (HA oligos) have been shown to upregulate anabolic gene expression, MMP-2 and MMP-9 synthesis and activation in vitro. *MMP1* and *MMP13*, *ADAMTS1*, *ACAN*, *COL1A1* and *COL2A1* gene expression were all up-regulated in an annular lesion model of DDD and promoted annular repair processes [31]. A 16-amino acid fragment of link protein (LinkN, DHLSDNYTLDHDRAIH) has been shown to promote matrix synthesis by the resident disc cell population [32,33,34,35] and stimulated MSC proliferation and differentiation and promoted IVD repair [36,37]. Peniel 2000 is 2 kDa biglycan derived peptide with the ability to inhibit TGF-β1 activity and has provided beneficial effects in the treatment of disc degeneration [38]. Resveratrol (3,5,4′-trihydroxy-*trans*-stilbene), a plant phenolic compound found in the skins of grapes, blueberries, and raspberries, also has beneficial properties with regard to the treatment of DDD [29,39,40]. A number of commonly prescribed anti-cholesterol lipid lowering medications have also produced beneficial effects on disc cell metabolism of application in the treatment of DDD. Simvastin [28,41,42], ortovastatin, and lovastatin [41,42,43,44,45] display protective effects on the IVD. A flavonoid isolated from grapefruit (naringin) and component of the traditional Chinese herb *Rhizoma drynariae* (Gusuibu) [46] has potent anti-inflammatory and anti-oxidant properties [47] which upregulates NP cell proliferation and down-regulates tumour necrosis factor (TNF)-α activity but elevates BMP-2, aggrecan and type II collagen protein production and also upregulates *ACAN* and *SOX6* and decreases *MMP3* gene expression [48]. This indicates that naringin may be a useful therapeutic agent in the treatment of DDD. A number of studies have evaluated the use of mesenchymal stromal stem cells (MSCs) for the treatment of DDD [26,49,50,51,52,53,54] and a number of reviews have covered this area of repair biology [55,56,57,58,59,60]. The mode of action of how MSCs illicit their therapeutic response in-situ remains an unanswered question and this needs to be addressed before MSCs can be morally advocated as a routine therapeutic intervention for DDD. A recent study using MSCs isolated from vertebrae demonstrated that they exhibited paracrine effects in co-cultures with AF and NP cells [61]. MSCs down-regulated pro-inflammatory cytokine gene production in degenerate NP (IL-1α, IL-1β, IL-6, and TNF-α) and AF cells (IL-1α and IL-6) and promoted extracellular matrix deposition. Growth factor mRNA was also elevated in MSC co-cultures, epidermal growth factor (EGF) insulin-like growth factor (IGF)-1, osteogenic protein (OP)-1, growth and differentiation factor (GDF)-7 and transforming growth factor (TGF)-β were all up-regulated by NP cells and IGF-1, OP-1 and GDF-7 by AF cells. These therapeutic effects are in keeping with the use of growth factor therapy to induce biological repair of degenerate IVDs [62,63] and establishes a paracrine mode of action for MSCs.

The EuroDISC clinical trial [64] utiliszd expanded autologous disc cells in single level discectomy patients, 28 patients reported greater pain reduction at 24 months than a control group and their IVDs had increased fluid contents evident by magnetic resonance imaging (MRI). Percutaneous injection of expanded autologous MSCs in two non-controlled clinical trials has also demonstrated improved MRI T2 signal and clinical improvement [65,66]. Use of autologous bone marrow derived MSCs in two small groups of DDD patients resulted in clinical improvement in 9/10 patients while conservative treatment failed [65,66]. A phase II clinical trial conducted with adult bone marrow MSCs for the treatment of back pain [67] resulted in the majority of the treated patients achieving a significant reduction in LBP. This has led to a multi center Phase III MSC clinical trial in DDD patients in 25 centers throughout the USA.

A major hindrance in the assessment of the efficacy of such biological interventions has been the inadequacy of many of the small animal models so far developed to examine DDD (reviewed in [68,69]). Large animal models of DDD have been developed in sheep [70,71,72], goats [73,74,75], dogs [76,77,78], and pigs [79,80,81] and, of these, the sheep represents the gold standard large animal model. In 1990, Osti and colleagues developed a model of DDD induced by controlled anterolateral (5 × 5 mm) surgical defects over a 24 months post operative (PO) period [71]. A number of studies have used this model and demonstrated spatiotemporal changes in discal and paradiscal components such as the NP [72], cartilaginous end plates (CEPs) [82], facet joints [83] and vertebral bone adjacent to and distant from the lesion site [84], ingrowth of blood vessels and nerves [85], focal expression of fibroblast growth factor (FGF)-2, TGF-β1 and α-smooth muscle cell actin [86] by cell populations associated with annular remodeling and repair of the lesion site. In an effort to develop a more aggressive model and to minimize maintenance costs, a modified ovine large lesion model was developed in 2012 using a 6 × 20 mm lesion [70]. The mechanical destabilization so induced led to more rapid lesion development in the AF and degeneration of the NP over a three to 6 month PO period rather than the 18–24 months required in the Osti model [72]. With a 6 × 20 mm lesion, qRT-PCR gene profiling demonstrated an elevation in catabolic matrix gene (*MMP2*, *MMP3*, and *MMP13*; and *ADAMTS4* and *ADAMTS5*) expression, these features have also been described in pathological human IVDs [87,88,89,90] and shown to severely impact on the biomechanical competence of the IVD. In the present study we compared the histopathological features of DDD and developed a quantitative scoring scheme. The efficacy of MSCs in the promotion of discal repair could also be evaluated using this scoring scheme.

## 2. Results

**Figure 1:**
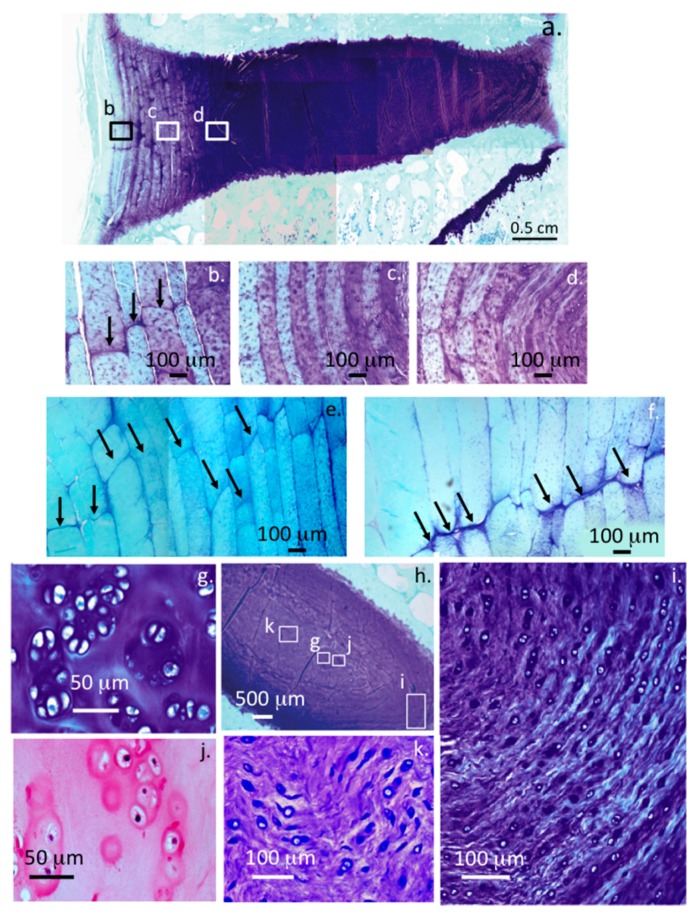
A Toluidine blue-fast green stained macroscopic view in vertical views of 4 µm sections of a two-year-ovine IVD (**a**); the boxed areas b, c, and d are also presented at higher magnification to provide detail of the tissue organization and cellular morphologies (**b**–**d**); translamellar cross-bridge formations (arrows) in the outer and inner AF of a three-year-old ovine IVD (**e**,**f**) stain positively with toluidine blue; isolated small clones of chondroid cells are also occasionally evident centrally in the NP (**g**,**h**,**j**); while normal NP cells are single (**k**); occasional cell doublets are evident in the inner AF (**i**).

Figure 1 depicts proteoglycan staining with toluidine blue/fast green in a sham operated two-year-old ovine lumbar IVD, in 4 µm vertical sections demonstrating normal disc structure and proteoglycan levels. The specific areas of interest are represented by the boxed areas in the macro view of an entire IVD and adjacent vertebral body segments at the top of the figure composite are also shown at higher magnification in Figure 1a–c. Proteoglycan staining is typically faint in the outermost annular lamellae (Figure 1a) but the intensity of metachromatic proteoglycan staining (purple) increases in the mid (Figure 1b) and inner lamellae and is maximal in the NP (Figure 1c). Alternate annular layers in the mid and inner AF are discernable due to differences in staining intensity with toluidine blue and its merging with the NP (Figure 1d). Translamellar cross bridges also stain up with toluidine blue, indicating there is proteoglycan deposition in these structures (arrows) which provide interconnectivity between annular layers (Figure 1e,f). The cells are dispersed singly throughout the annular layers and are discernable as typical fibrochondrocytes, occasional doublet cells and small strings of cells arranged in arcade like arrangements along the lines of fibrillar lamellar collagens in the transitional zone where the inner AF lamellae merge with the NP (Figure 1c).

**Figure 2:**
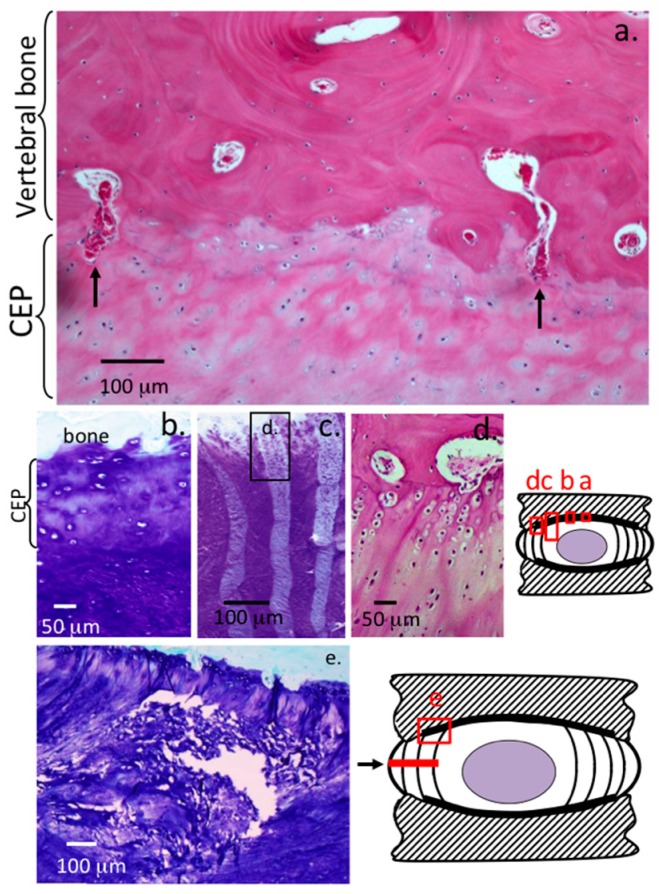
Histological detail of the cartilaginous endplate (CEP) and adjacent vertebral bone and annular attachment regions in a 4 µm thick vertical sections of normal lumbar three-year-old ovine IVD. H&E stained section with two blood vessels penetrating through the CEP arrowed (**a**); a similar region of the central NP CEP is depicted stained with toluidine blue (**b**); the CEP of the mid AF with annular attachments alternately stained (**c**); and a higher power view of a central region in (**c**) depicted as an H&E stained section in (**d**); an area of cystic degeneration adjacent to the central CEP (**e**) is also depicted and the area of the disc affected indicated in a disc schematic (**e**). The cartoons depict the areas of interest in the IVD shown elsewhere in this figure. Abbreviations: BV, blood vessel; CEP, cartilaginous endplate. Segments (**c**,**d**) modified from [91].

Proteoglycan staining is maximal in the NP as evident in the low power view provided in Figure 1a. The morphology of the NP cells are typical of the small fibrochondrocytic observed elsewhere in the IVD however occasional small chondroid cell nests are discernable in some cases these differ from the typical resident NP cells (Figure 1k) since they are frequently seen to be dividing in these nests and are surrounded by a more extensive dense and non-fibrous basophilic matrix (Figure 1g,j). Furthermore, while very little fibrous material is in evidence in such chondroid cell nests, these are a prominent feature around the resident NP cells (Figure 1k). Detail of the cellular arrangements in the NP margins/transitional zone demonstrate the presence of some doublet cells and small strings of interconnected fibrochondrocytes laid down in arcade type patterns following the distribution of fibrillar lamellar collagens (Figure 1i). Proteoglycans are also heavily stained with toluidine blue in the transitional zone (Figure 1i). The CEP contains cells of a rounded chondrocyte-like morphology in a proteoglycan rich matrix typical of a hyaline cartilage (Figure 2a,b) and can be differentiated from the adjacent IVD tissues where fibrous material is more prominent and the cells are smaller. H&E stained samples of CEP clearly depict the demarcation between CEP and adjacent vertebral bone, occasional blood vessels penetrating the CEP into the vertebral body are a normal feature and serve as a nutritional route for IVD cells (Figure 2a). The AF attaches to the CEP by a series of fibrillar attachments into the vertebral bone (Figure 2c,d). Cystic degeneration was also evident in a few IVDs at the margins of the NP/CEP where proteoglycans were depleted and cells were dead (Figure 2e).

**Figure 3:**
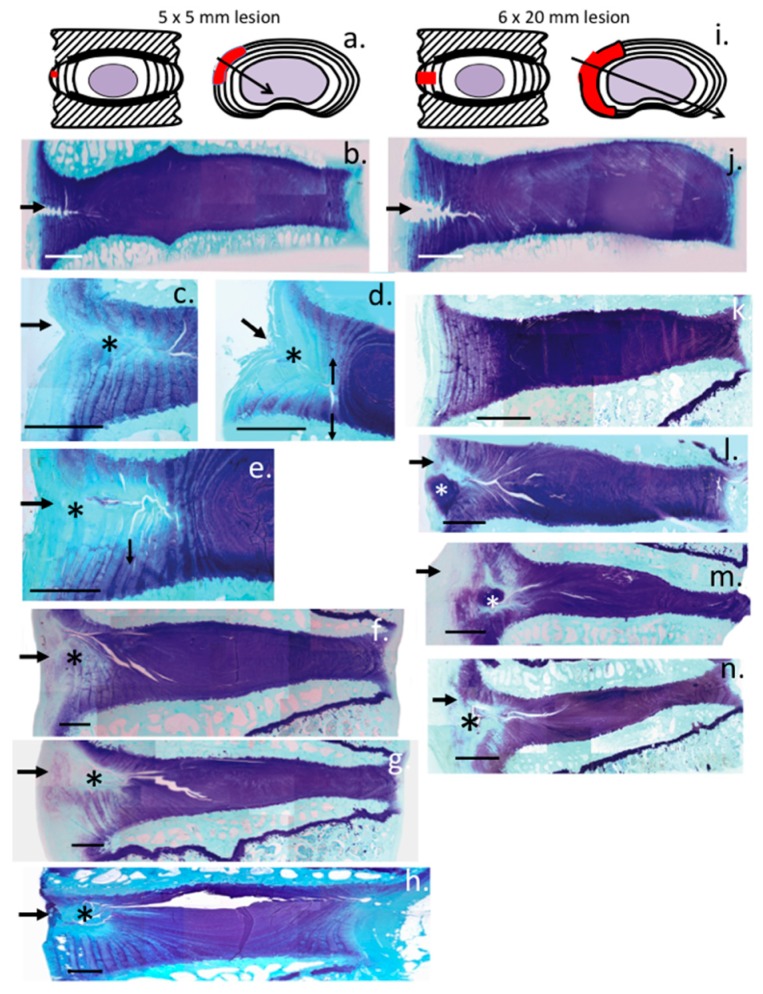
Histological appearance of IVDs affected by transverse outer annular lesions (arrows) in 4 µm vertical sections taken perpendicularly through the lesion zone. The sections taken are indicated in the schematics at the top of the figure with an arrow running through the lesion site depicting the oblique tissue sections sampled through the lesion site. Schematics depict IVDs and the 5 × 5 mm (**a**); and 6 × 20 mm lesion (**i**) used in this study, with the extent of the experimental defects indicated by colored red areas; Proteoglycan localization by toluidine blue-fast green staining are shown in vertical oblique sections of IVDs at various time-points post-lesion induction in the: 5 × 5 mm (**b**–**h**); and 6 × 20 mm lesion (**k**–**n**) DDD models; Toluidine blue/fast green stained vertical oblique sections of two-year-old (**b**–**h**) lumbar ovine IVDs. The arrows on the left hand side of each photosegment depict the original lesion site; Segment (**b**) is a non-operated control (NOC) disc in which a 5 × 5 mm lesion had been made just prior to histology; Segment (**j**) is a NOC disc in which a 6 × 20 mm lesion had been made; The 5 × 5 mm lesion IVD sections sampled: three months post-operation (PO) (**c**,**d**); six months PO (**e**); twelve months PO (**f**); eighteen months PO (**g**); and twenty-four months PO (**h**). Areas labeled with a black asterisk in (**c**–**h**) indicate focal areas of proteoglycan depletion. Notice the significant reduction in disc height twelve to eighteen months PO in lesion IVDs (**f**,**g**) and partial recovery and propagation of the defect over time into the contralateral AF (**h**). Three months post operative 6 × 20 mm lesion IVDs are also presented for comparison (**l**–**n**); and a NOC disc is presented in (**k**) for comparison. White asterisks in (**l**,**m**) represent areas of chondroid metaplasia, and the black asterisk in (**n**) represents focal proteoglycan depletion. Scale bars in the histology images are 500 µm.

Schematic depictions of the 5 × 5 and 6 × 20 mm lesions in vertical and plan view (Figure 3a,i) and how the oblique sections of the lesion site perpendicular to the lesion zone were sampled. A 5 × 5 mm deep (Figure 3b) and 6 × 20 mm (Figure 3j) anterolateral transverse lesion cut in the outer AF of a cadaveric IVD followed by immediate histological processing including toluidine blue staining showed the appearance of these lesions without allowing any time for them to propagate further into the AF (Figure 3b,i). Longitudinal assessment of the 5 × 5 mm lesion discs over three to twenty-four months PO (Figure 3c–h) demonstrated a focal loss of proteoglycan in the outer AF lesion site at three months PO (Figure 3c,d); by six months PO (Figure 3e), the clearing of proteoglycans from the lesion site and inversion of the annular lamellae was more pronounced and small defects propagating from the original lesion were evident including delaminations and the start of circumferential tear development (Figure 3e). Lesion development was even more advanced at 12 and 18 months PO (Figure 3f,g), again focal loss of proteoglycan from the lesion site was a prominent feature and a reduction in disc height and toluidine blue staining in the NP was also evident. By 24 month PO, the lesion had now developed through the NP to the contralateral AF, focal loss of proteoglycans in the outer AF of the original lesion site was still evident (Figure 3h). The relative size and location of the antero-posterolateral 6 × 20 mm IVD lesion is represented schematically in Figure 3i, and shown in a freshly cut and processed cadaveric disc (Figure 3j), compared to a non-operated control (NOC) disc (Figure 3k). There was no focal loss of toluidine staining in the lesion site since this is a time dependent process mediated by the cells in the lesion site, which are subject to alterations in the biomechanical micro-environment which modulates cell gene expression. Examination of the proteoglycan staining in the lesion affected IVDs three months PO revealed focal depletion of proteoglycans from the outer AF and propagation of the lesion into the NP (Figure 3l–n). A reduced disc height was also clearly evident and focal chondroid metaplasia of the outer AF (Figure 3l,m). Reduced proteoglycan staining was also evident in the NP of the 6 × 20 mm lesion affected IVDs three months PO (Figure 3l–n).

**Figure 4:**
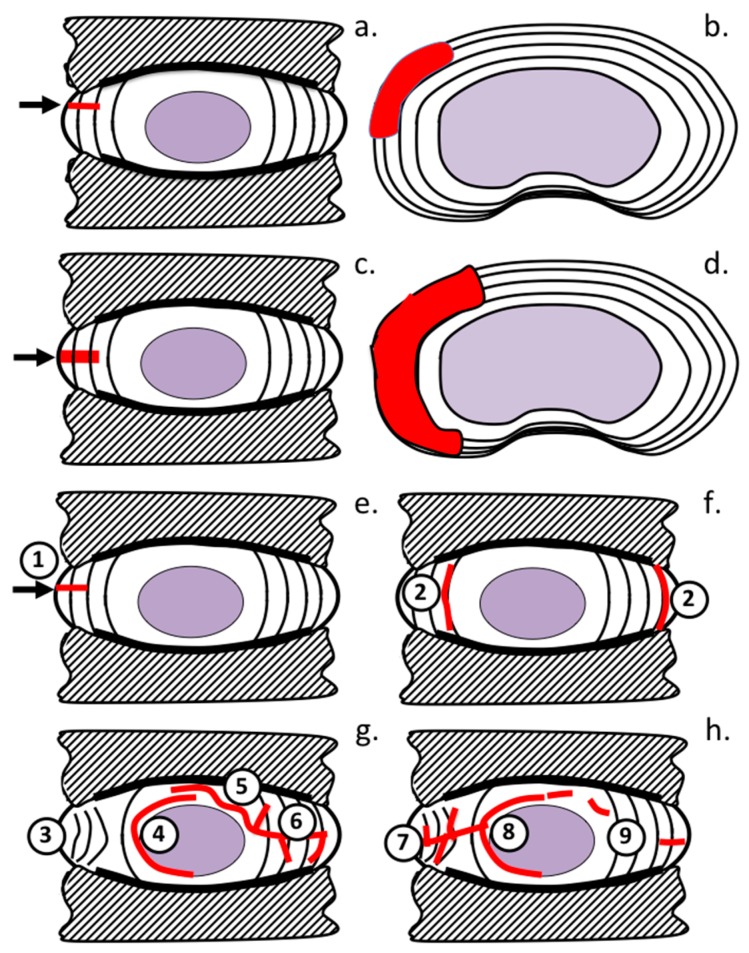
Schematic depiction of anterolateral lesion types used to initiate experimental IVDD: 5 × 5 mm lesion (**a**,**b**); and 6 × 20 mm lesion (**c**,**d**). A summary of the time dependent changes in lesion morphology in the: 5 × 5 mm lesion (**e**–**g**); and 6 × 20 mm lesion models (**h**) with lesions indicated in red. These include: rim lesions (1); radiating tears (2); annular inversion (3); propagation of radiating tears around NP (4); and into contralateral AF (5); development of delaminations (6); early development of delaminations in outer AF in 6 × 20 mm lesion (7); and propagation of radial tears around NP (8); and into contralateral AF (9).

A schematic diagram of the 5 × 5 mm (Figure 4a,b) and 6 × 20 mm lesion (Figure 4c,d) and how these propagate through the IVD over time was constructed to summarize these changes (Figure 4e-h).

**Figure 5:**
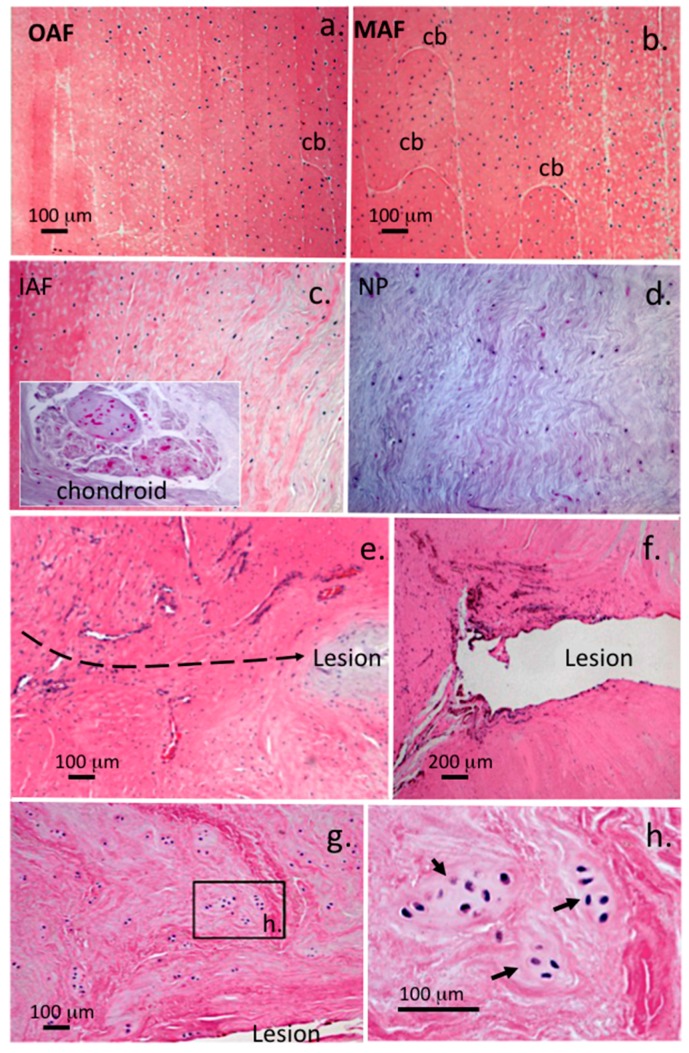
Comparison of the cellular distributions and morphologies in the: outer (OAF) (**a**); mid (MAF) (**b**); inner AF (IAF) (**c**); and NP (**d**) of a NOC ovine lumbar IVD compared to two examples of lesion affected IVD specimens stained with H&E (**e**,**f**); four micron thick vertical sections are depicted, with areas of the transverse lesion in (**e**–**g**); an influx of blood vessels and cells associated with the 5 × 5 mm lesion twelve month PO (**e**) and the unhealed inner AF in a 6 × 20 mm lesion three months PO (**f**); cell cloning adjacent to the lesion site was evident in the 6 × 20 mm lesion discs (**g**,**h**). The boxed area in (**g**) is presented in higher magnification in segment (**h**). Small chondroid cell nests were also occasionally observed in the NOC NP however in the example shown (panel **c** insert) these cells appeared to be dead. Abbreviations: cb, translamellar cross bridge. Scale bars shown are 100 μm, plates **c** and **d** are shown at the same magnification as plates **a** and **b**. The dotted line in e depicts the propagation plane of the lesion.

A re-examination of the cellular morphologies at medium power magnification in the outer, mid and inner AF and NP of NOC IVDs stained with H&E showed a relatively disperse collection of single cells, doublet cells were also seen but only very occasionally in the inner AF (Figure 5a–d). Occasional chondroid cell nests were also observed in the NP however in the case shown in Figure 5 (insert) most of the cells were dead. These resembled the cell nests reported in Figure 1g,j. The antero-lateral annular lesions in the 5 × 5 mm lesion at 12 months PO (Figure 5e) and the 6 × 20 mm lesion at three months PO (Figure 5f) contained a large influx of cells and blood vessels. Small cell clusters were also observed near the 6 × 20 mm lesions (Figure 5g,h) similar to the chondroid cell nests previously described in the NP of NOC IVDs (Figure 1g,j).

**Figure 6:**
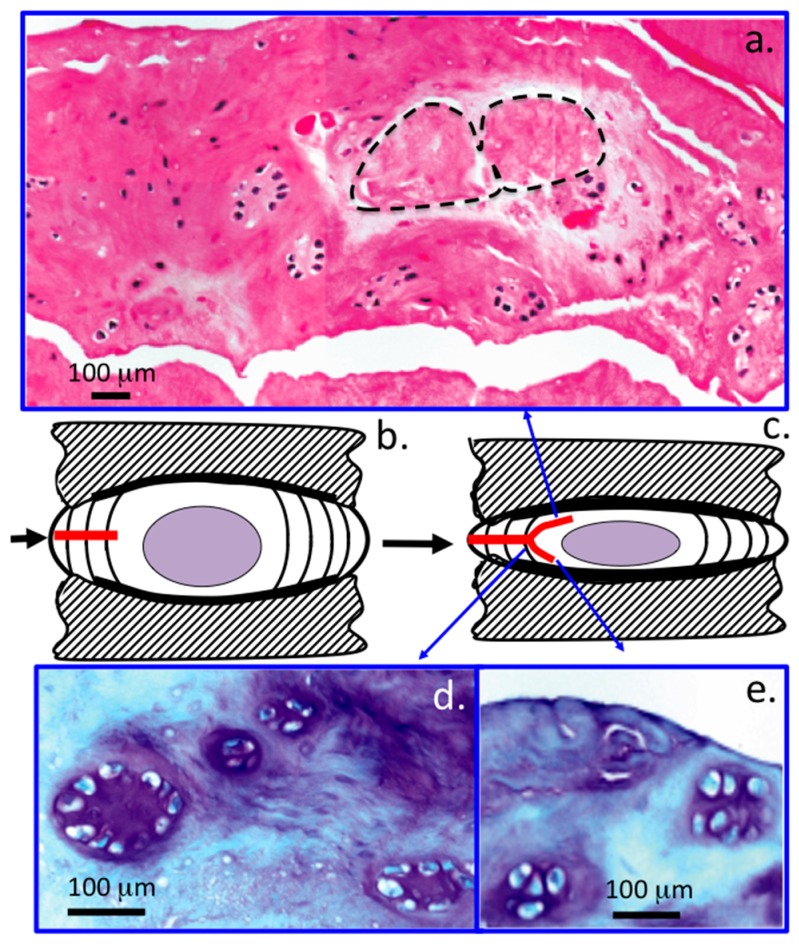
Histological assessment of cellular morphologies in ovine IVDs in: H&E (**a**); and toluidine blue-fast green stained tissue sections, 4 µm sections (**d**,**e**) in a 6 × 20 mm lesion at three months post lesion induction; schematic depictions of the lesion affected IVD are also shown in (**b**,**c**) showing the initial lesion site and its propagation by three month post surgery; small clones of cells are clearly evident in the vicinity of the annular lesion (**a**,**d**,**e**). Areas of tissue necrosis surrounded by dotted lines are also apparent in (**a**). Segment (**d**) is modified from [70].

A closer inspection of the lesion associated cellular populations around the 6 × 20 mm lesions in higher power images (Figure 6a,d,e) confirmed that small groups of cell nests in discrete basophilic matrices were located in close proximity to the non-healed annular lesions that had propagated into the inner AF and NP margins (Figure 6a,d,e), similar less prominent chondroid cell nests had previously been observed in some of the NOC NP specimens (Figure 1g,j). The largest cell nests in the lesion affected AF tended to be in areas where maximal proteoglycan depletion was also evident (Figure 6d,e). These cellular morphologies were not a prominent feature of the Osti lesion affected IVDs but were evident in the 6 × 20 mm lesion IVDs in the vicinity of the annular lesions (Figure 6a). These IVDs are also depicted schematically (Figure 6b,c). The NOC ovine IVD also contained a relatively sparse distribution of singlet fibrochondrocytes throughout the outer AF (Figure 1b and Figure 5a), mid AF (Figure 1c and Figure 5b), inner AF (Figure 1d and Figure 5c) and NP (Figure 1k and Figure 5d).

**Figure 7:**
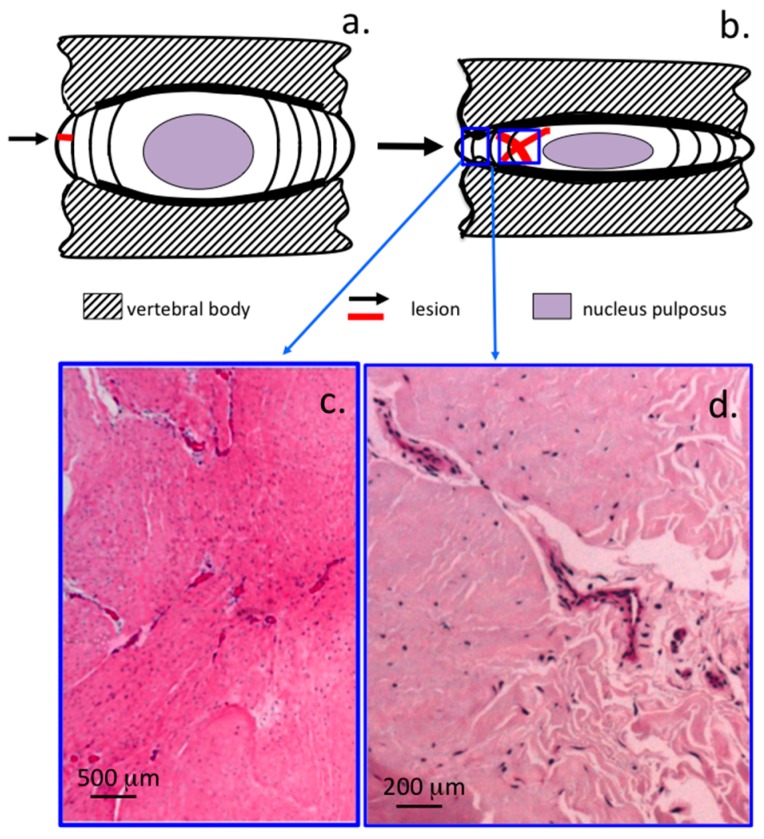
Diagrammatic representation of 5 × 5 mm anterolateral lesion affected IVD at: three (**a**); and six months (**b**) post lesion induction in 4 µm thick vertical IVD sections. Hematoxylin and eosin stained vertical sections of the AF are also shown of the blue colored boxed areas indicated in (**b**); in photosegments (**c**,**d**), an influx of blood vessels in the outer AF has undergone partial healing (**c**); however the lesion is still clearly evident in the inner AF (**d**).

**Figure 8:**
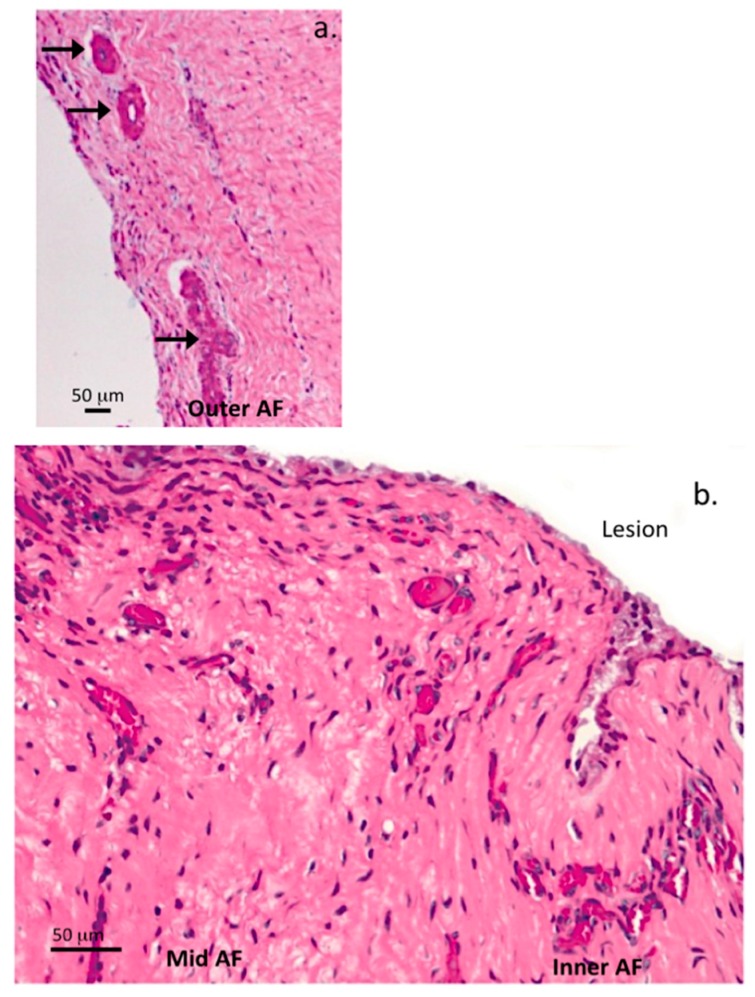
Visualization of blood vessels in transverse lesion affected IVDs, vertical 4 µm thick IVD sections. H&E stained vertical tissue sections through a lesion site outer AF, 5 × 5 mm lesion 6 months PO (**a**); and inner AF adjacent to a lesion site 5 × 5 mm lesion twelve month PO (**b**), demonstrating a few large prominent blood vessels (arrows) in the outer AF, a moderate influx of cells along the lesion track (**a**) and large influx of blood vessels with pink stained entrapped red blood cells and heavy infiltration of cells along the lesion site in the inner AF (**b**).

**Figure 9:**
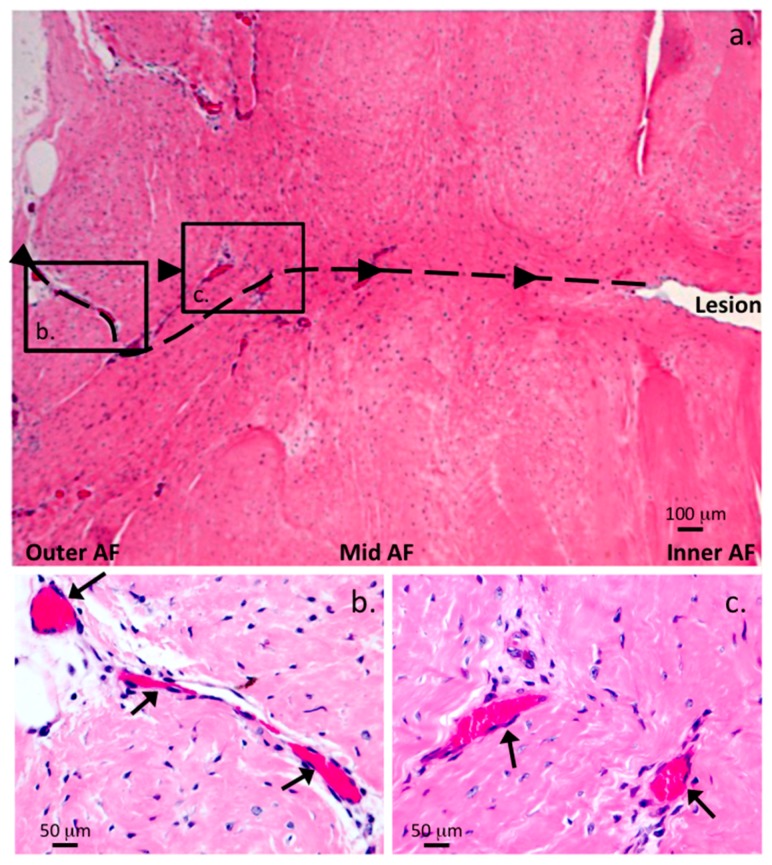
Immunohistochemical visualization of blood vessels and cellular influx into the 6 × 20 mm annular lesion site in 4 µm vertical IVD sections. Low power H&E stained vertical IVD section demonstrating the numerous blood vessels and cellular influx throughout the AF three months PO. Higher power views of two selected boxed areas in (**a**) are also presented showing the vessels (arrows) and entrapped red blood cells stained bright pink (**b**,**c**). A prominent influx of fibroblastic cells throughout the outer lesion site repair tissue is also a prominent feature in the AF but less so around the non-healed inner lesion site in this specimen. The propagation pathway of the lesion is depicted with a dotted line in (**a**), boxed areas are depicted at higher magnification below. The dense pink stained regions are entrapped red blood cells which intensely take up the H&E stain.

An influx of blood vessels was also a prominent feature of the 5 × 5 mm (Figure 7a,b and Figure 8a,b) and 6 × 20 mm annular lesions (Figure 9a–c).

In the early stages of disc degeneration between 3–6 months PO in the 5 × 5 mm lesion discs blood vessels were prominent in the outer AF (Figure 7c,d) but were very rare in age matched normal IVDs, when rarely seen in NOC IVDs, the blood vessels rarely penetrated past the outermost collagenous lamellae. By 12 months post induction of the annular lesion, blood vessels had penetrated throughout the AF to the unhealed lesion site in the inner AF (Figure 8b). The 6 × 20 mm annular lesions had a similar ingrowth of blood vessels and associated cell populations to those seen in the 12 months 5 × 5 mm lesions; however, in the 6 × 20 mm lesion model, this occurred by three months PO (Figure 9a–c).

Many of these vessels contained entrapped red blood cells confirming their identity, fortuitously the red blood cells stained a prominent pink color with the H&E stain and could be easily identified. By 24 months in the 5 × 5 mm lesions, this cellular influx and blood vessels had regressed and the cell distribution in these tissues now resembled those of age matched IVDs.

**Figure 10:**
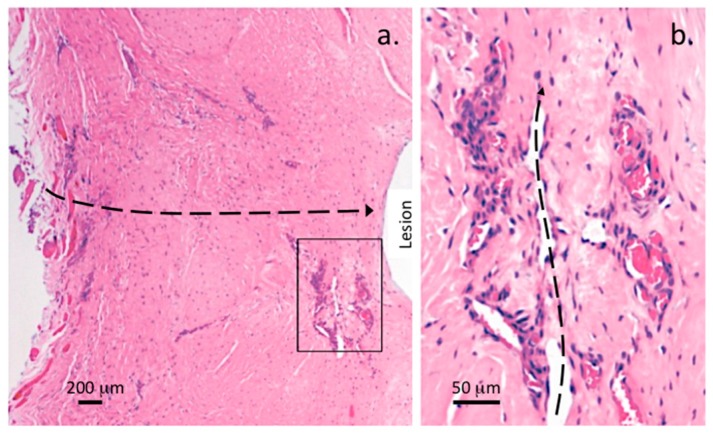

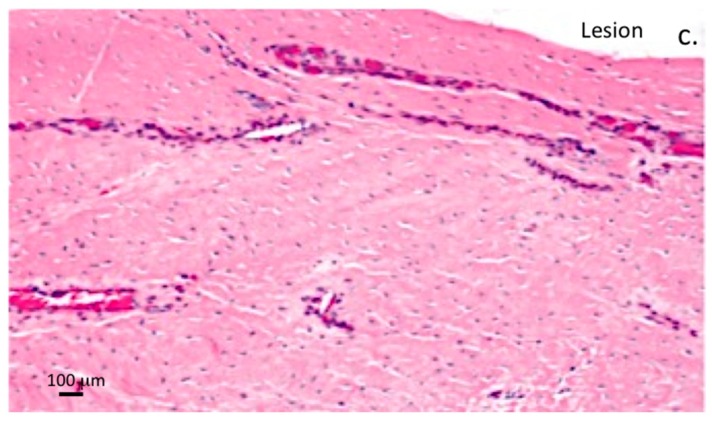
Low and medium power H&E stained 4 µm thick vertical disc sections through two lesion sites depicting prominent blood vessels in the outer AF with entrapped red blood cells stained prominently and the major influx of fibroblastic cells throughout the lesion site of a 6 × 20 mm lesion affected IVD (**a**) and prominently associated with vessels adjacent to the non-healed lesion in the inner AF, boxed area in (**a**); presented at higher magnification (**b**); A prominent longitudinally sectioned blood vessel (arrows) associated with a non-healed lesion in the mid AF is also evident with a moderate influx of cells around the lesion (**c**). The dotted lines in (**a**,**b**) depict the original pathway of the outer lesion (**a**) or vascularized unhealed inner lesion (**b**).

The 6 × 20 mm lesion affected IVDs had a significant influx of cells and blood vessels into the AF (Figure 10a) and around the lesion site (Figure 10b,c) resembling that evident in the 5 × 5 mm lesion IVDs at 12 months PO (Figure 8b).

**Figure 11:**
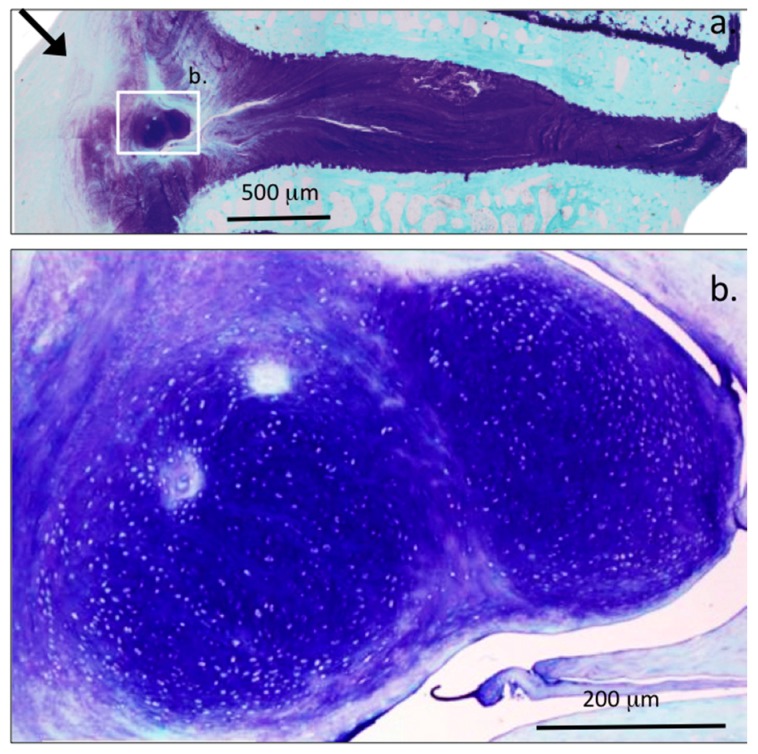
Toluidine blue histology of a 4 µm thick vertical disc section demonstrating proteoglycan localization (**a**); and a region of chondroid metaplasia (**b**) associated with the annular lesion site in a 6 × 20 mm lesion affected IVD at six months PO demonstrating high levels of proteoglycan production by cells of a rounded chondrocyte-like morphology. The arrow in (**a**) depicts the original site of the outer annular lesion.

Focal loss of proteoglycan around the penetrating lesion was evident in the inner AF (Figure 11a) however occasional areas of intense toluidine blue staining due to chondroid metaplasia and focal proliferation of chondrocyte like cells (Figure 11b) was also evident in some cases. This contrasted with the small cell clusters in areas of the proteoglycan depleted lesion in the inner AF.

**Figure 12:**
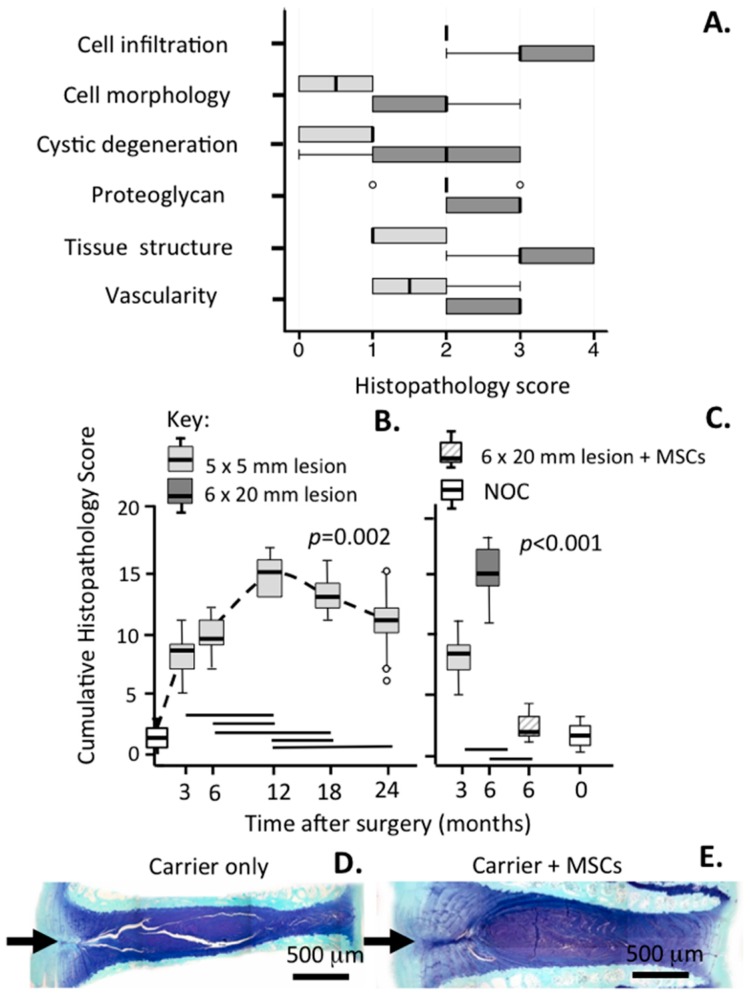
Histopathological scoring of IVDs from the 5 × 5 mm and 6 × 20 mm lesion models of experimental disc degeneration. The range of individual parameters which were scored for the cumulative histopathology score are indicated in (**A**); Longitudinal scores for the 5 × 5 mm lesion model up to 24 months post surgery are indicated in (**B**) and in the 6 × 20 mm lesion in (**C**). Overall, the time points for appearance of DDD are significantly different (Kruskal–Wallis rank test; *p* = 0.002 for **B** and *p* < 0.001 for **C**). A significant reduction in the cumulative histopathology score in the 6 × 20 mm lesion model from 15.7 to 2 was evident after administration of MSCs following a three-month period with an anterolateral lesion to induce degeneration and a three-month recovery period with MSCs (**C**). This was statistically significant (*p* = 0.001). NOC = non operated control. Histological sections of lesion affected discs stained with toluidine blue-fast green are depicted in (**D**,**E**). In (**D**), the lesion disc was injected with PBS carrier without stem cells; In (**E**), stem cells were injected; Notice the prominent lesion in (**D**) and significantly reduced disc height compared to (**E**) where a near normal disc height and almost complete disappearance of the lesion is evident, this disc however still had to attain the staining intensity of a NOC disc. The arrow in (**D**,**E**) depicts the original lesion site. For comparisons with a NOC disc see Figure 1a or Figure 3k. The bars above the *x* axes in (**B**,**C**) indicate significant differences between time points (Mann–Whitney *U* test; *p* < 0.05). In the box plots the centre line is median, box is 25%/75%; whiskers are 10%/90%; dots are outliers.

The histopathology scoring scheme outlined in Table 1 was applied to disc sections from the 5 × 5 mm and 6 × 20 mm lesion models (Figure 12A–C). This showed differences between the two models in each of the discriminative parameters used in the histopathology scoring scheme and the more aggressive disc degeneration induced by the 6 × 20 mm lesion (Figure 12A). Administration of MSCs into lesion discs where DD had been induced three months earlier had followed by a three-month recuperative period resulted in a dramatic resolution of the degenerative features in these lesion discs. This was evident as a drop in the cumulative histopathology score from 15.7 to 2.0, close to the score for NOC IVDs and was statistically significant *p* = 0.001 (Figure 12C). Histology plates in Figure 12 clearly show that PBS carrier alone was an ineffective therapeutic agent with the annular lesion propagating through to and around the NP a very significant reduction in disc height was also evident (Figure 12D). The MSC injected IVDs, however, re-attained a near normal disc height and the annular lesion was barely discernable, a significant therapeutic achievement reflected in a decrease of the histopathology degeneracy score from 15.7 to 2.0 (Figure 12E).

## 3. Discussion

Adams and Roughley proposed a useful definition of IVD degeneration as *an aberrant, cell-mediated response to progressive structural failure* [22]. Nociceptive nerve in-growth into degenerate human IVDs contribute to the perception of LBP [92] in ~40% of degenerate IVDs [25]. A recent ten-year global study, which surveyed 291 major human diseases, placed LBP as the number one musculoskeletal disorder [93]. Approximately 80% of the general population in Western Societies is affected by LBP and its incidence increases with aging, peaking in the fifth and sixth decade [93]. In 1998, UK costings for the treatment of LBP were costed at £12.3 billion [94], in 2001 LBP cost $9.17 billion in Australia [95]. In 2006 the American Academy of Pain Medicine published costs for chronic pain in the USA at $560–635 billion/annually, 53% of all chronic pain patients had LBP and 31 million people were estimated to suffer from LBP at any one time [25,30,96]. In 1999, the World Health Organization (WHO) published the IRIS low back pain initiative [97] which highlighted LBP as a priority area and designated the development of biomaterials for disc replacement and stem cell methodology to restore functional IVDs as high priorites [26]. LBP was also made a National Research Priority Area by the National Health and Medical Research Council (NHMRC) and Australian Institute of Health and Welfare (AIHW) in 2009 [96] but to date have failed to fund any innovative projects designed to alleviate or better understand this condition. This is a major deficiency given the major and ever-increasing impact LBP makes on the Australian community [95,98]. Recent findings of the global burden of disease study 2010 [93] found that, of the 289 major human diseases examined, LBP ranked highest in terms of overall disability in terms of disability adjusted life years (DALYs) confirming the impact of LBP on the daily life of all individuals [99,100,101]. A number of animal models and preclinical studies have demonstrated the utility of MSCs for disc repair [37,61,69,102,103,104,105]. Phase I and II clinical trials with MSCs have already been completed and a phase III clinical trial is currently underway. In the USA.

Magnetic Resonance Imaging (MRI) shows ~40% of the general population display radiographic evidence of DD, a significant proportion of these patients suffer from LBP [106,107] suggesting that these conditions are causally linked [108,109,110]. LBP arising from DDD may be caused by inflammatory responses triggered by degenerate IVD tissue herniating into the spinal canal [111], nerve ingrowth into the IVD [110,112] or due to altered spinal biomechanics [113]. In terms of pain relief and recovery of spinal function, surgery (spinal fusion) remains the most effective treatment currently available but is associated with degenerative IVD changes adjacent to the fusion mass. Biological methods are also of interest as a treatment option [27,58,114,115,116,117,118]. A major hindrance to the assessment of the efficacy of such therapeutic strategies has been the inadequacy of the animal models available to examine DDD (reviewed in [68]). Large animal models of DDD have been developed in dogs [76,77,119], sheep [70,71,72], goats [73,74,75] and pigs [79,80,81]. The latter two species suffer from anomalously high spontaneous healing responses and persistence of notochordal cells in the IVD with ageing, compounding unequivocal interpretation of the efficacy of therapeutic repair strategies. Sheep and canine represent appropriate animal models for such evaluations relevant to the human condition. Spinal manipulation studies demonstrate annular lesions influence IVD biomechanics, neurophysiological responses, vertebral lumbar motion segment responses, muscular contributions to dynamic dorsoventral lumbar spinal stiffness and three-dimensional vertebral motion in lumbar spinal motion segments. Multifidus muscle remodeling [33,120,121,122,123,124,125,126,127,128] in response to DDD may also be a source of LBP. Changes in multifidis pro-inflammatory cytokine gene expression [127] occur in response to experimental DDD [70]. Similar changes in human spinal tissues and LBP have been reported with DDD [129] reinforcing the appropriateness of the ovine model for valid comparisons with the human spine.

Disc cells obtain their energy primarily by anaerobic glycolysis to generate adenosine triphosphate and lactic acid from glucose [130]. An adequate supply of glucose is an absolute requirement for disc cell nutrition. Disc cells die within 2–3 days under low glucose conditions [131]. Diffusive processes from peripheral blood vessels are a major nutritional pathway for disc cells and for the removal of metabolic waste products (lactate). Oxygen tensions of 5.8 KPa in the CEPs and outer AF and 2 KPa in the central NP in human IVDs have been measured. Lactate concentration gradients inversely mirror IVD O_2_ tension profiles ranging from 2 mmol/L in the outer AF to 6 mmol/L in the center of the NP [132,133,134]. Lactic acid concentrations in the disc can reach 6–8 mM compared to 1 mM in plasma, leading to an acidic pH of 6.9–7.1 in IVDs. With DD, lactic acid concentrations can reach 20 mM and intradiscal pH values ~6.0 [135,136]. Matrix synthesis in the IVD ceases if the pH falls below 6.8 [136] compromising disc cell viability and an impairment in disc tissue homeostasis [137,138,139,140,141,142,143,144]. This effect is more pronounced under conditions of low glucose concentrations (<0.5 mM). Degenerate IVDs with a pH < 6.5 are unsuitable candidates for repair using MSCs since at these pH values reduced cell proliferation, viability, an absence of matrix synthesis and lowered anabolic gene expression is evident. The pH in a healthy IVD varies between 7.0–7.2, but falls to 6.7–6.9 in a mildly degenerate IVD. Severely degenerate IVDs have a pH of 6.0–6.5. A pH below 6.8 in the IVD shuts down MSC cell proliferation and matrix production, thus it is important to determine the degenerative status of candidate IVDs to determine their suitability for MSC treatment. Improvements in IVD imaging facilitate the non-invasive determination of the degenerative status of an IVD based on its internal pH and glycosaminoglycan (GAG) profile and aids in patient selection [145,146].

In 1990, Osti and colleagues were awarded the Volvo Prize for the development of a model of DDD using controlled anterolateral (5 × 5 mm) surgical defects which induced DDD over a 12–18 month post operative (PO) period [71]. A number of studies using the Osti model have demonstrated that annular defects also impact on other discal and paradiscal components such as the NP [72], CEPs [82], facet joints [83] and vertebral bone adjacent to and distant from the lesion site [84]. The ingrowth of blood vessels and nerves [85] into experimentally injured IVDs and focal expression of FGF-2, TGF-β1 and α-smooth muscle cell actin [86] by cell populations associated with the lesion sites have also been demonstrated. The impact of lesion progression on disc proteoglycans has demonstrated degradation of aggrecan [147], upregulation of decorin and biglycan [148] and fragmentation of biglycan and fibromodulin [149]. In an effort to develop a more aggressive model of DDD in sheep a 6 × 20 mm annular lesion was used to induce DDD by three months post surgery rather than the 12–18 months PO in the Osti model [70]. The resultant mechanical destabilization induced de-lamellation, radial lesions, and bifurcation of the outer annular lesion, and anomalous modification of annular attachments to the CEP [150] similar to those identified in the Osti model [71] and in human DDD [87,90]. Altered discal biomechanics (Neutral zone, forward and lateral bending, torsion, range of motion) and qRT-PCR gene profiling of selected representative anabolic and catabolic matrix genes further demonstrated the utility of this model which closely reproduced defects described in pathological human IVDs [87,88,89,90]. These background studies were the impetus for the development of the histopathological scoring scheme described in the present study. This six-category scheme examined PG depletion, changes in annular structure and cellular morphology associated with experimental DD. The ingrowth of blood vessels and cellular influx into annular defect sites, chondroid metaplasia and cystic degeneration provided additional quantitative information on DD. This scheme was also useful for assessing changes in degenerate IVDs induced by administration of MSCs into a degenerate IVD resulting in a reduction in total histopathology score from 17.5 to 2.2 over a three-month recuperative period. This was a significant finding considering the large size of the degenerative lesion in this model and clearly demonstrated the regenerative power of MSCs.

A review of existing classification schemes for human DD published in PubMed from 1957–2016 uncovered 42 schemes, 30 of these dealt with the lumbar spine. Progressive stages of spinal degeneration affecting the IVDs, facet joints, vertebral bodies and spinal canal were described in these studies (reviewed in [151]). Five of these grading schemes used microscopic anatomy, four used histology, six used plain radiography and five MRI while three utilized discography to describe degenerative changes in spinal components. Other classification schemes have used combinations of these techniques plus biomechanics [152], MRI, biochemical composition, histology, radiography [153], or MRI and discography to score DDD [154,155]. Existing imaging methods such as X-ray, CT and MRI can reliably detect changes in the IVD with degeneration however they lack enough sensitivity to reliably detect the earliest stages of DDD. Novel MRI techniques such as magnetization transfer spinal imaging [156,157,158,159], chemical exchange saturation [145,146], ultrashort echo time MRI, and sodium MRI [160,161,162,163,164,165] are sensitive to ECM molecules such as proteoglycans and collagens and these newer imaging modalities hold promise in the detection of quantitative alterations in functional discal ECM components in the early stages of DDD [166,167]. Patients with identifiable early stages of DDD likely to progress to a chronic stage are expected to be the most suitable candidates for biological therapeutic interventions to improve discal structure and function.

Kellgren and Lawrence (1957) [168] were the first to use radiography to classify degenerative changes in the osteoarthritic spine with grade 1 representing minimal anterior osteophytosis, grade 2 definite anterior osteophytosis and possible disc space narrowing and sclerosis of the vertebral plates. Grade 3 was characterised by moderate narrowing of the disc space, vertebral plate sclerosis and osteophytosis. Grade 4 described severe disc space narrowing, sclerosis of vertebral plates and multiple large osteophytes. The Kirkaldy-Willis classification scheme of 1978 [169] considered degenerative changes including spinal dysfunction, herniation, instability, lateral nerve entrapment, single and multiple level central spinal stenosis. Adams et al. (1986) [170] classified, stages of DD using discography and described, “cottonball” IVDs as those with no signs of AF degeneration but a soft white amorphous NP; “lobular” discs were a mature disc with the NP starting to coalesce into fibrous lumps; “Irregular”, a degenerative disc with fissures and clefts in the NP and inner AF; “fissured” IVDs were degenerative discs with radial fissures stretching from the inner AF to the outer edge of the AF and “ruptured” IVDs contained a complete radial fissure allowing fluid to escape the IVD. Pathria et al. (1987) [171] developed a radiographic scheme to grade facet joint disease into 4 degenerative stages. Grade 0, normal; grade 1, facet joint space narrowing; grade 2, facet joint narrowing plus sclerosis or hypertrophy; grade 3, severe OA of the facet joint with narrowing, sclerosis and osteophytes. In 1988, Modic et al. [172,173] used T1 and T2 MRI to describe marrow changes in the vertebral bodies associated with DDD and categorized these into three stages (Modic type I–III). Type I, decreased T1 signal but increased T2 signal representing marrow edema associated with disruption and fissuring of the endplate and vascularized fibrous tissue within the adjacent marrow. Modic Type II, increased T1 signal and iso-intense or hypo-intense T2 signal due to fatty degeneration of subchondral marrow, endplate disruption with yellow marrow replacement in adjacent vertebral body. Modic Type III, decreased T1 and T2 signal correlated with extensive bony sclerosis on plain X-rays, dense woven bone with no marrow to produce MRI signal. MRI assessment of Modic changes due to vertebral bone lesions, indicated fatty marrow infiltration and inflammatory edematous changes in vertebral bone [174]. Thompson et al. (1990) [175] evaluated radiographic, morphologic and anatomical assessments of DDD and went on to correlate these with MRI and zonal IVD compositional changes. Weiner et al. (1994) developed a four-point classification scheme correlating radiographic spinal changes with alterations in spinal flexibility, progressive degenerative changes in facet joints and IVDs based on the extent of disc space narrowing, osteophyte formation and bone eburnation. Weishupt et al. (1999) [176] used MRI and CT to assess facet joint changes associated with DDD and developed a 4 point grading scheme based on facet joint narrowing, osteophyte development, hypertrophic changes in the articular processes, subarticular bony erosions and subchondral cysts. Pfirrmann et al. (2001) [177] used MRI to assess spinal degeneration concentrating on changes in the IVDs. The Pfirrmann scheme consists of 5 grades. Grade I, IVD homogeneous with a bright hyper-intense white signal intensity and a normal disc height. Grade II, IVD structure inhomogeneous with a hyper-intense white signal but clear distinction between NP and AF and normal disc height with or without horizontal grey bands. Grade III, IVD structure inhomogeneous with an intermediate grey signal intensity, unclear distinction between NP and AF, disc height normal or slightly decreased. Grade IV, IVD structure inhomogeneous, hypo-intense dark grey signal intensity, no distinction between NP and AF, disc height normal or moderately decreased. Grade V, IVD structure inhomogeneous, hypo-intense black signal intensity, no distinction between NP and AF, disc space collapsed. Carragee et al. (2003) [178] classified disc degeneration in terms of lumbar disc herniation classified into: (i) “fissure type”, a herniation with minimal annular defect and extruded or sequestrated fragment; (ii) “defect type” herniation with a large or massive annular defect and an extruded or sequestrated fragment; (iii) ”contained type”, herniation with an intact AF and one or more sub-annular detached fragment; and (iv) “no-fragment, contained type” herniation with an intact AF and no sub-annular detached fragment. Thalgott et al. (2004) [179] developed a six-point classification system for DDD based on a combination of MRI, provocative discography, plain X-rays and specific anatomic features which assessed spinal curvature and a 4 point assessment of facet joint degeneration. Most recently, Wang and colleagues (2012) [180] categorized changes in vertebral endplates and correlated these with age and the onset of DDD. In 2013 Rutges and colleagues (2013) [181] published a validated histological classification scheme to describe the various stages of human DDD. This is similar to a classification scheme for canine disc degeneration [76] and to the scheme we have developed in the present study but is less discriminative.

A number of studies have documented positive results with MSCs in disc repair [37,49,65,182,183,184,185,186,187] and this has also been the subject of a number of definitive reviews [26,58,188,189,190,191]. The results we obtained with MSCs in the ovine annular lesion model of experimental DD are therefore consistent with these earlier findings. Furthermore, the histopathological scoring we undertook of control and MSC treated tissues in the present study further reinforced the efficacy of MSCs as a therapeutic agent and the validity of the scoring system.

Intervertebral disc degeneration involves changes in many of its constituent tissues [22] and any prospective scheme aimed at scoring histopathological changes in this composite connective tissue must address each of these components and herein lies a major strength of the proposed new scoring scheme. While useful, most of the previously developed classifications of DDD are incapable of precisely identifying details of all of these aspects of DDD although recent improvements in X-ray, MRI and d-GEMRIC technology is correlating imaging modalities with supportive biochemical data [192]. Annular rim-lesions due to fatigue failure of the Sharpey fiber attachments of the annulus into the vertebral body rim are a common feature in DDD and these lesions may propagate into the inner AF and even through to the contralateral AF in extreme cases [21]. Bifurcation of the rim lesion due to separation of adjacent annular lamellae in the mid and inner AF are also evident in DD and may lead to development of radial fissures. These would be expected to severely impact on the ability of the AF to withstand the hoop stresses which it normally resists during axial spinal loading, and may actually be the driving forces which propagate annular lesions [193]. Focal loss of proteoglycan around annular lesions is also found in DDD and demonstrate changes in local catabolic processes by the resident disc cell populations presumably in response to changes in their biomechanical microenvironment produced by annular lesions [70]. An influx of cells to the annular lesion site occurs in IDD, some of these express TGF-β and FGF-2 [86] and these growth factors have roles in promoting the laying down of ECM components by the resident disc cells. FGF-2 is also an angiogenic growth factor and promotes the ingrowth of blood vessels into lesion affected degenerate IVDs [85]. Cell clustering also occurs in such regions of the IVD affected by annular lesions and may be a response by the resident stem cell populations [70]. Changes in the CEP (including cyst formation) can also contribute to DD through disruption in normal nutrient pathways to the disc cells [26]. Loss of proteoglycan from the NP in DD leads to a reduction in the ability of this tissue to resist compression and a reduction in disc height [194,195]. Taken together, it is clear that DDD is a multifactorial disorder and a histopathological scoring scheme must reflect this. The proposed scheme should be suitable for the description of DDD in any large animal and human IVD. Use of this scheme may further identify that like degenerative arthritic changes in OA and RA [196,197,198,199,200,201,202,203], DDD displays a spectrum of degenerative features and several sub-types of DDD are likely to be identified in the future when sufficient data is collected for critical evaluation. However for such a development to occur one must have a reliable, discriminative scoring scheme capable of evaluating the relative importance of each of the listed degenerative features of DDD. It is our contention that the proposed new classification scheme we have developed and described herein provides such a scheme.

## 4. Materials and Methods

The data reported in this study is based on studies conducted in 1992 [72] and 2012 [70] where 5 × 5 and 6 × 20 mm annular lesions were used to induce DDD in merino wethers, all methodology is provided in detail in these earlier publications. Ethical approval for conducting all animal procedures was obtained from the Royal North Shore Hospital Animal Care and Ethics Committee of The University of Sydney in 1992 and in 2011 as outlined in earlier studies [70,72].

### 4.1. Ovine Models of DDD

Briefly, merino wethers aged 3–4 years of age were purchased at local sale yards. Sixty-four animals were used in the 5 × 5 mm lesion study, animals were randomly separated into 4 groups of 16 animals, 8 of the animals in each group received controlled anterolateral lesions in the L1L2, L3L4 and L5L6 IVDs, the remaining animals did not receive a lesion and served as sham operated controls (surgical approach only). All animals were maintained in open pasture and allowed to roam freely post surgery. The animals were subsequently sacrificed at 6, 12, 18 and 24 months post operatively (PO) and the spines removed (two additional sheep were also sacrificed 3 month PO). The IVDs of interest and adjacent vertebral body segments were isolated using a bone saw and fixed in 10% neutral buffered formalin. In the 6 × 20 mm lesion study group thirty-two merinos were separated into a lesion group and sham operated group. The Lesion group received 6 × 20 mm anterolateral lesions in the L1L2, L3L4 and L5L6 IVDs. Animals were sacrificed 3 month PO and the spines were excised and IVDs of interest isolated for histology.

### 4.2. Induction of Disc Degeneration and Administration of Mesenchymal Stem Cells

A total of 18 three-year-old merino wether sheep were divided into 3 groups (lesion plus two non-operated control (NOC) sheep groups). All of the animals (except NOC sheep) received 6 × 20 mm annular lesions at the L1L2, L3L4 and L5L6 spinal levels by our established model [70]. After 12 weeks to establish disc degeneration, a second surgery was performed to expose the contralateral AF away from the lesion site and 10 million MSCs in 0.2 mL PBS or PBS carrier were injected into the NP through the undamaged AF of the L1L2, L3L4 and L5L6 lesion IVDs. After a further 14 weeks recovery from surgery the sheep were sacrificed and the lumbar IVDs collected for histological examination.

### 4.3. Isolation and Characterization of Mesenchymal Stem Cells

Details of the isolation and demonstration of pluripotency of ovine mesenchymal stem cells from pooled iliac crest bone marrow are as noted earlier [204]. Frozen cell stocks were thawed from nitrogen storage and grown in monolayer culture for several passages to expand cell numbers, passage 8 cells were used in this study. The chondrogenic, adipogenic and osteogenic pluripotency of these MSCs was confirmed as indicated earlier prior to their use in the annular lesion model [204].

### 4.4. Histological Processing of IVDs

Prior to fixing the IVDs, all soft tissue surrounding the IVDs was trimmed off, the posterior elements were removed with bone cutters and most of the superior and inferior vertebral bodies adjacent to each IVD were trimmed off with bone saws. The remaining bone was trimmed down to ~1 mm from the margins of the IVD using a Dremel burring tool. En-bloc fixation of the IVDs was undertaken for 48 h in 10% neutral buffered formalin. The vertebral body margins attached to the IVD are essential for the preservation of native tissue architecture and prevention of artifactual swelling of IVD specimens during fixation [205]. However, it is also critical to minimize the amount of bone attached to the IVD specimens to cut down on the decalcification time for each specimen to ensure the preservation of cellular detail and antibody epitope reactivity for later immunolocalization studies. Decalcification was undertaken with 10% formic acid 5% neutral buffered formalin with constant agitation Vertical tissue blocks (~5 mm thick) were taken of the IVD-vertebral bodies perpendicular to the lesion sites. These tissue blocks were dehydrated in graded ethanols and xylene and embedded in paraffin wax. Due to the large size of the 6 × 20 mm lesion this was sampled at three sites across the lesion site to obtain representative samples and these were separately evaluated. Four-micron vertical tissue sections were cut and adhered to SuperFrost Plus microscope slides for histological examination. Tissue sections were de-paraffinized in two changes of xylene prior to rehydration of the slides through graded ethanol washes (100–70%) to water.

### 4.5. Histochemistry

#### 4.5.1. Toluidine Blue Staining

Vertical sections (4 µm) of IVD and superior and inferior vertebral bodies were stained for ten minutes with 0.04% *w*/*v* toluidine blue in 0.1 M sodium acetate buffer, pH 4.0, to visualize the glycosaminoglycans followed by a two minute counterstain in 0.1% *w*/*v* fast green FCF.

#### 4.5.2. Hematoxylin and Eosin Staining

Tissue sections were stained in Mayers Hematoxylin (five minutes), rinsed in tap water blued in Scotts Blueing solution (one minute) and counterstained in 0.0001% eosin (5 min), dehydrated in 95% ethanol then absolute ethanol, cleared in xylene and mounted.

### 4.6. Development of a Histopathological Scoring System for IVD Specimens

An extensive 27 point six category histopathological scoring scheme (See Table 1) was developed based on: (i) toluidine blue proteoglycan localization; (ii) lesion structural characteristics; (iii) cellular morphology; (iv) extent of blood vessel ingrowth; (v) cell infiltration; and (vi) presence or absence of specific degenerative features such as cystic degeneration or chondroid metaplasia. These parameters were assessed in the outer, mid and inner AF, NP and contralateral AF of the lesion affected and sham operated IVDs.

### 4.7. Statistics

Power calculations from previous studies suggested there was a 90% power of detecting a four unit difference in histology score using six sheep per group. Histology scores were tested for significance using the Kruskal–Wallis (KW) equality-of-populations rank test in the first instance. If significance was found (*p* < 0.05), individual groups were compared using the Wilcoxon rank-sum test. Within each parameter, the Benjamini–Hochberg post hoc test was used to correct for false positives. All statistical analyses were performed using STATA 13 statistical software (Survey Designs, StataCorp LP, College Station, TX, USA). Data are presented as box plots with median values indicated with a solid horizontal bar

## 5. Conclusions

The histopathological scoring scheme described in the present study can be employed to quantitatively score IVD degeneration and regeneration following administration of adult MSCs and should also be applicable to the assessment of the efficacy of other biological interventions. Furthermore, this scheme can be used in conjunction with imaging (X-ray and MRI) and functional methods such as biomechanics and with biochemical compositional and gene expression techniques to examine these processes using a highly informative multidisciplinary approach.

## Figures and Tables

**Table 1:** Histopathological scoring of normal and pathological ovine IVDs. Abbreviations: IAF, inner AF; MAF, mid AF; OAF, outer AF; NP, nucleus pulposus; CEP, cartilaginous endplate.

Grade	Histopathological Features
**A. Toluidine Blue/Fast Green Proteoglycan Staining**	
0	Fast green staining only of OAF, metachromatic Toluidine blue staining of IAF, intense metachromatic staining in NP, well defined CEP staining. Alternate AF lamellae discernable due to differing metachromatic staining intensities of adjacent lamellae
1	Moderately reduced metachromatic staining of MAF/IAF in vicinity of lesion, fast green staining of OAF only, normal metachromatic staining of NP and CEP
2	Reduced patchy metachromatic staining around lesion, fast green staining in OAF (no metachromasia)
3	Reduced metachromatic staining in NP compared to sham or NOC IVD, very faint or no metachromatic staining in OAF/MAF, fast green staining only in OAF
**B. IVD Structure/Lesion Characteristics**	
0	Normal IVD structure with well defined annular lamellae, central NP and CEP
1	Lesion evident in MAF, normal NP morphology
2	Lesion evident in MAF/IAF, lesion but may not be apparent in OAF due to spontaneous repair, IAF lamellae may be inverted and have anomalous distortions in normal lamellar architecture
3	Bifurcation/propagation of lesion from MAF/IAF into NP margins, mild delamination, when more extensive may lead to concentric tears between lamellae in MAF/IAF
4	Propagation of lesion into NP, with disruption in normal NP structure, distortion of annular lamellae into atypical arrangements-severe delamination, separation of translamellar cross bridges
5	Lesion reaching through NP into contralateral posterior AF with disruption in normal NP structure
**C. Cellular morphology**	
0	Normal, sparse distribution of typical single AF and NP fibrochondrocytes
1	Small groups of rounded chondrocytic cells (two to four cells/group) in vicinity of annular lesion in IAF, occasional cell division in resident inner AF and NP cells
2	Moderate increase in well defined groups of rounded dividing cells (four to eight per group) in vicinity of lesion and with penetrating blood vessels associated with the lesion site, well defined chondroid cell colonies in NP contained within a dense basophilic matrix with little fibrillar material evident around the cells contrasting with NP cells
3	Numerous cell clones around IAF/MAF lesion, chondroid cell nests in NP containing >50 cells
**D. Blood Vessel Ingrowth**	
0	Very occasional vessels in outermost annular lamellae, occasional capillaries in CEP
1	Occasional blood vessels in OAF and MAF
2	Moderate number of blood vessels in IAF
3	Extensive ingrowth of vessels in IAF/MAF and along lesion margins
**E. Influx of cells into the lesion site**	
0	Normal cell distribution in OAF, MAF, IAF and NP
1	Slight influx of cells mainly in outer AF
2	Moderate influx of cells throughout AF
3	Large influx of cells throughout AF
4	Heavy influx of cells throughout AF particularly in inner AF and around lesion
**F. Histolopathological features not covered elsewhere**	
2	Chondroid metaplasia in AF
2	Cystic degeneration affecting ≥5% NP
3	Extensive cystic degeneration affecting ≥20% NP
4	“Bare” fibrillar elements in NP due to loss of ground substance, confirmed by a paucity of toluidine blue metachromasia affecting ≥20% of NP and also evident as a reduced disc height
